# Phosphorylation of mRNA-Binding Proteins Puf1 and Puf2 by TORC2-Activated Protein Kinase Ypk1 Alleviates Their Repressive Effects

**DOI:** 10.3390/membranes11070500

**Published:** 2021-06-30

**Authors:** Henri A. Galez, Françoise M. Roelants, Sarah M. Palm, Kendra K. Reynaud, Nicholas T. Ingolia, Jeremy Thorner

**Affiliations:** 1 Department of Molecular and Cell Biology, Division of Biochemistry, Biophysics and Structural Biology, University of California, Berkeley, CA 94720, USA; henri.galez@etu.utc.fr (H.A.G.); roelants@berkeley.edu (F.M.R.); s_palm@berkeley.edu (S.M.P.); ingolia@berkeley.edu (N.T.I.); 2 Graduate Group in Biophysics, University of California, Berkeley, CA 94720, USA; kendra.reynaud@berkeley.edu

**Keywords:** cell signaling, plasma membrane homeostasis, *Saccharomyces cerevisiae*, *Pumilio* family RNA-binding proteins

## Abstract

Members of the Puf family of RNA-binding proteins typically associate via their *Pumilio* homology domain with specific short motifs in the 3’-UTR of an mRNA and thereby influence the stability, localization and/or efficiency of translation of the bound transcript. In our prior unbiased proteome-wide screen for targets of the TORC2-stimulated protein kinase Ypk1, we identified the paralogs Puf1/Jsn1 and Puf2 as high-confidence substrates. Earlier work by others had demonstrated that Puf1 and Puf2 exhibit a marked preference for interaction with mRNAs encoding plasma membrane-associated proteins, consistent with our previous studies documenting that a primary physiological role of TORC2-Ypk1 signaling is maintenance of plasma membrane homeostasis. Here, we show, first, that both Puf1 and Puf2 are authentic Ypk1 substrates both in vitro and in vivo. Fluorescently tagged Puf1 localizes constitutively in cortical puncta closely apposed to the plasma membrane, whereas Puf2 does so in the absence of its Ypk1 phosphorylation, but is dispersed in the cytosol when phosphorylated. We further demonstrate that Ypk1-mediated phosphorylation of Puf1 and Puf2 upregulates production of the protein products of the transcripts to which they bind, with a concomitant increase in the level of the cognate mRNAs. Thus, Ypk1 phosphorylation relieves Puf1- and Puf2-mediated post-transcriptional repression mainly by counteracting their negative effect on transcript stability. Using a heterologous protein-RNA tethering and fluorescent protein reporter assay, the consequence of Ypk1 phosphorylation in vivo was recapitulated for full-length Puf1 and even for N-terminal fragments (residues 1-340 and 143-295) corresponding to the region upstream of its dimerization domain (an RNA-recognition motif fold) encompassing its two Ypk1 phosphorylation sites (both also conserved in Puf2). This latter result suggests that alleviation of Puf1-imposed transcript destabilization does not obligatorily require dissociation of Ypk1-phosphorylated Puf1 from a transcript. Our findings add new insight about how the TORC2-Ypk1 signaling axis regulates the content of plasma membrane-associated proteins to promote maintenance of the integrity of the cell envelope.

## 1. Introduction

Signaling pathways can enable rapid responses to changing external conditions via phosphorylation of proteins that directly influences their activity, localization and/or stability. Accompanying these more immediate regulatory events, signaling can concomitantly elicit longer term adaptation through effects on gene expression because transcription factors are often among the targets of phosphorylation. Further fine-tuning of stimulus-evoked changes in protein expression also can be achieved via phosphorylation of RNA-binding proteins that regulate the packaging, transport, sequestration, localization, translation efficiency, and rate of decay of mRNAs [1,2,3,4,5]. 

In the genome of budding yeast (*Saccharomyces cerevisiae*), there are approaching 700 gene products annotated as RNA-associated proteins [6,7]. Among those that specifically bind to mRNAs are six members of the highly conserved PUF family of mRNA-binding proteins [8,9,10]: Puf1/Jsn1, Puf2, Puf3, Puf4, Puf5/Mpt5 and Puf6. The Puf nomenclature derives from the RNA-binding domain shared by its first two recognized members, Drosophila *Pumilio* (PUM) and *Caenorhabditis elegans fem-3*-binding factor (FBF) [11,12]. 

In a global screen we conducted to identify potential substrates of the protein kinase Ypk1 [13], we pinpointed both Puf1/Jsn1 and its paralog Puf2 (46% identity; 56% similarity) as likely candidates. Puf1 contains two consensus Ypk1 phospho-acceptor sites and Puf2 contains four (and the two sites present in Puf1 occur at the same relative positions as the corresponding two sites in Puf2). Puf1 and Puf2 are localized at the cell periphery [14,15,16]. Moreover, the preponderance of the client transcripts that associate with Puf1 and Puf2, as determined either by affinity isolation using TAP-tagged derivatives of each protein [15] or via crosslinking-immunoprecipitation (CLIP) methodology [17], are mRNAs that encode plasma membrane (PM)-associated proteins, including integral PM proteins. In addition, it was reported [18] that puf1 and puf2 loss-of-function mutations each conferred some degree of increased resistance of yeast cells to the antibiotic myriocin, which is a potent and specific inhibitor of L-serine:palmitoyl-CoA acyltransferase (SPT) [19], a heterotrimeric (Lcb1-Lcb2-Tsc3) endoplasmic reticulum (ER)-localized enzyme that catalyzes the first reaction in sphingolipid biosynthesis. That same phenotype (increased resistance to myriocin) is observed when Ypk1 function is elevated because Ypk1 action upregulates flux through the sphingolipid pathway at two steps: by phosphorylating and thereby alleviating inhibition of SPT by its endogenous negative regulators, the ER-localized tetraspanins Orm1 and Orm2 [20]; and, by phosphorylating and thereby stimulating the activity of the catalytic subunits (Lac1 and Lag1) of ceramide synthase, a heterotrimeric ER-localized enzyme (also containing Lip1) [13]. In yeast, as in all eukaryotes, the final destination for the bulk of cellular sphingolipids is the external leaflet of the PM [21,22].

Ypk1 is the only essential downstream effector of the PM-associated Target of Rapamycin Complex 2 (TORC2) because a hyperactive allele of Ypk1, Ypk1(D242A), is able to bypass loss-of-function mutations in all known TORC2 subunits [20,23,24]. In normal cells, the extent of TORC2-mediated phosphorylation of Ypk1 at a series of five C-terminal sites [25] adjusts its activity so that the cell is able to cope appropriately with stresses and insults that have the potential to damage or compromise the integrity of the PM (reviewed in [26,27]). Ypk1 has a paralog (Ypk2); however, Ypk1 is, by far, the major isoform under standard growth conditions (30 °C) [28] because the *YPK2* gene is only highly expressed under heat shock conditions [29,30].

Aside from stimulating sphingolipid biosynthesis via its phosphorylation of Orm1, Orm2, Lac1 and Lag1, as mentioned above, other well-characterized substrates of Ypk1 control other cellular processes necessary for the maintenance of the PM, including modulation of the supply of glycerol for glycerophospholipid synthesis [31,32], regulation of the leaflet distribution of aminophosphoplipids [33] and the content of ergosterol [34,35], and adjustment of the rate of endocytosis of integral PM proteins [36,37,38,39,40].

Given the recovery of Puf1 and Puf2 in our proteome-wide screen for additional Ypk1 substrates, and the prior biochemical, cytological, and phenotypic indications that Puf1 and Puf2 are connected to PM components, we sought in this study to confirm that Puf1 and Puf2 are authentic targets of Ypk1-mediated phosphorylation, to validate that phosphorylation of Puf1 and Puf2 by Ypk1 has significant physiological consequences, and to elucidate at the mechanistic level how Ypk1 phosphorylation influences Puf1 and Puf2 function.

## 2. Materials and Methods

### 2.1. Construction of Yeast Strains and Growth Conditions

*S. cerevisiae* strains in this study (listed in Table 1) were constructed, in the main, using standard methods for genetic manipulation of yeast [41]. Insertion of an in-frame 3XFLAG tag appended to the coding sequence of *PMP3* was accomplished by Cas9-mediated gene editing, using the method described elsewhere [42]. Yeast strains were grown routinely at 30 °C on standard rich (YP) or defined minimal (SC) media containing 2% glucose as carbon source and supplemented with appropriate nutrients to select for the retention of plasmids, when present.

### 2.2. Plasmids and Recombinant DNA Methods

All plasmids in this study (listed in Table 2) were constructed using standard procedures in *Escherichia coli* (*E. coli*) strain DH5α [43]. All constructs were verified by nucleotide sequence analysis in the UC Berkeley DNA Sequencing Facility. All PCR reactions were performed using Phusion^TM^ High-Fidelity DNA Polymerase (New England Biolabs Inc.). Site-directed mutagenesis was performed using appropriate mismatch oligonucleotide primers and the QuikChange^TM^ method (Agilent Technologies, Santa Clara, CA, USA), according to the manufacturer’s instructions.

### 2.3. Purification of GST-Fusion Proteins

*E. coli* BL21(DE3) cells were transformed with plasmids expressing the desired proteins or protein fragments as fusions to the C-terminus of GST in plasmid pGEX4T. Cells were grown overnight in LB medium with ampicillin for plasmid selection in a 37 °C water bath. The next day, cultures were diluted to A600 nm = 0.2, grown at 30 °C to A600 nm = 0.6, induced by the addition of isopropyl β-D-1-thiogalactopyranoside (IPTG) (final concentration 0.6 mM), and incubated for a further 4h at 16 °C before harvesting by centrifugation. Cell pellets were rinsed with ice-cold 1XPBS and snap-frozen by immersion in liquid N_2_ and stored at −80 °C before use. For protein purification, the frozen cell suspensions were thawed on ice and diluted into 10 mL ice-cold PBS lysis buffer (PBS, 0.5% Tween-20, 1 mM EDTA, 2 mM MgCl_2_) per 50 mL of the original culture into which was dissolved one cOmplete™ protease inhibitor tablet (Roche). To commence lysis, lysozyme (final concentration 0.2 mg/mL) was added and, after incubation on ice for 20 min, the cells were ruptured on ice by two 15-sec bursts of sonication with a 1-min interval of cooling in between each burst. To the resulting lysate, dithiothreitol (DTT) was added (final concentration 1 mM) and the lysate was subjected to centrifugation at 12,000× *g* for 10 min at 4 °C. The resulting clarified supernatant fraction was transferred to a fresh Falcon tube on ice containing 100 μL of a 50:50 slurry of glutathione-Sepharose 4B beads that had been pre-washed in ice-cold PBS lysis buffer. After incubation for 1 h on a lab tube rotator (LabQuake™, Thermo Fisher, Waltham, MA, USA) at 4 °C, the beads were collected by centrifugation, washed by three consecutive rounds of resuspension and recentrifugation in 1xPBS wash buffer (PBS, 0.1% Tween-20, 0.1 mM DTT) and the bead-bound GST-fusion proteins stored at 4 °C. To verify expression of the desired GST-fusion proteins, samples of the beads were analyzed by SDS-PAGE and staining with Coomassie blue dye. For use as substrate in protein kinase reactions, the beads with the bound GST-fusion protein were washed by two consecutive rounds of resuspension and recentrifugation in 1X kinase assay buffer (200 mM NaCl, 10 mM MgCl_2_, 0.1 mM EDTA, 50 mM Tris-Cl pH 7.5) and then eluted by resuspension with 80 μL of elution buffer (20 mM glutathione, 2 mM DTT, 1 mM Na_3_VO_4_ in 1X kinase assay buffer) followed by incubation for 15 min at room temperature on the lab tube rotator. The beads were removed by centrifugation and the resulting eluate transferred to a fresh Eppendorf tube. The quantity and purity of the purified GST-fusion protein was verified by SDS-PAGE and Coomassie staining.

### 2.4. Production and Purification of Ypk1-as from S. cerevisiae 

A mutant of Ypk1, Ypk1(L424A), that confers sensitivity to inhibition by the adenine analog 3-MB-PP1 (hereafter abbreviated Ypk1-as for “analog sensitive”) with a C-terminal His_6_-HA-3C-ZZ tag was expressed from the *GAL1* promoter on a *URA3*-marked multi-copy (2 µm DNA *ori*) plasmid (pAX50) in *ypk1-as ypk2*∆ cells (yAM135-A) [13], as follows. A starter culture was grown overnight in 50 mL of SC-Ura containing 2% raffinose and 0.2% sucrose and used to inoculate four 1L cultures in the same medium at an initial density of A_600 nm_ = 0.125. The cells were grown at 30 °C to A600 nm = 0.5 and then induced by addition of 100 mL 20% galactose to each culture. After further incubation for 18 h at 30 °C with vigorous aeration by gyratory shaking, the cultures were cooled on ice and the cells collected by centrifugation at 400 rpm for 12 min at 4 °C. The cell pellets were washed by one round of resuspension and recentrifugation in 10 mL 1X TAB-B* buffer (200 mM NaCl, 1.5 mM MgOAc, 1 mM DTT, 1x cOmplete Roche protease inhibitor tablet, 2 mM Na_3_VO_4_, 10 mM NaF, 10 mM Na-PPi, 10 mM β-glycerol-phosphate, 50 mM Tris-HCl, pH 7.5), resuspended in the residual liquid, and frozen by dispensing the cell suspension drop-wise into a kitchen strainer suspended in a well-insulated ice bucket containing liquid N_2_. The frozen cell beads were stored at −80 °C prior to use. Cell breakage was performed in a cryogenic cell disruptor (SPEX Sample Prep freezer mill, Model 6875); the frozen cell beads were placed in the mill and precooled in liquid N_2_ for 5 min and then ruptured by ten 2-min pulses of oscillatory grinding at 15 cps, with 1-min intervals of cooling between pulses. The resulting frozen powder of broken cells was transferred to a Falcon tube and stored at −80 °C prior to use. To initiate purification, the powder was thawed at room temperature, then placed on ice, and 2 vol of TAB-B* per g powder were added. After gentle mixing, the lysate was clarified by centrifugation in a SS-34 rotor for 20 min at 4 °C at 15,000× *g* in a refrigerated centrifuge (Sorvall). The resulting supernatant solution was transferred into 16 × 76 mm ultracentrifuge tubes and further clarified by centrifugation in a 50Ti rotor at 33,500 rpm at 4 °C for 1 h in an ultracentrifuge (Beckman L8-80M). Avoiding removal of the upper lipid cake, the remainder of the supernatant fraction was withdrawn with a long-tipped Pasteur pipette and transferred to a Falcon tube, adjusted to a final concentration of 0.15% NP-40, and then 1.25 mL of a 50:50 slurry of IgG-Sepharose beads (GE Healthcare) that had been prewashed with TAB-B2 buffer (200 mM NaCl, 1.5 mM MgOAc, 0.15% NP-40, 50 mM Tris-HCl pH 7.5) were added. After incubation for 1 h at 4 °C on a lab tube rotator, the slurry was transferred into a small glass column and the effluent liquid collected and passed over the bed of IgG-Sepharose beads again. To remove any unbound or non-specifically bound material, the bead bed was washed with four 5-mL portions of Post-IGG wash buffer (200 mM NaCl, 1.5 mM MgOAc, 1 mM DTT, 0.01% NP-40, 2 mM NaVO_4_, 10 mM NaF, 10 mM Na-PPi, 10 mM β-glycerol-phosphate, 50 mM Tris-HCl, pH 7.5). After draining the bead bed of any remaining liquid, it was washed with 5 mL of P3C/Ypk1 cleavage buffer (200 mM NaCl, 1.5 mM MgOAc, 1 mM DTT, 0.01% NP-40, 10% glycerol, 2 mM NaVO_4_, 10 mM NaF, 10 mM Na-PPi, 10 mM β-glycerol-phosphate, 50 mM Tris-HCl, pH 7.5). To elute the Ypk1-as enzyme from the beads, 30 µL PreScission™ protease (Genscript; 1 unit/µL) was diluted into 800 μL of P3C/Ypk1 cleavage buffer, then the protease solution was added to the drained beads in the column, which was sealed tightly with Parafilm™ and incubated overnight at 4 °C with gentle agitation on the lab tube rotator. After the cleavage reaction, the liquid in the column was drained into a siliconized Eppendorf tube. The PreScission™ protease comes as a fusion to GST and was removed from the column eluate by adding 10 µL of a 50:50 slurry of glutathione-Sepharose 4B beads that had been prewashed in P3C/Ypk1 cleavage buffer. After incubation for 2 h at 4 °C, the beads were removed by centrifugation at 4 °C in a microfuge at maximum rpm, and the resulting supernatant solution (containing the purified Ypk1-as) was removed, divided into aliquots, which were snap-frozen in liquid N_2_ and stored at −80 °C before use.

### 2.5. Protein Kinase Assay

Ypk1-as, purified as described in the preceding section, was incubated with the purified GST-fusion protein of interest in 1X kinase assay buffer (200 mM NaCl, 10 mM MgCl_2_, 0.1 mM EDTA, 50 mM Tris-Cl pH 7.5) with 100 µM ATP containing 2 μCi [γ-^32^P]ATP at 30 °C in the presence or absence of 10 μM 3-MB-PP1 in a total volume of 20 μL for 30 min. Reactions were terminated by the addition of SDS/PAGE sample buffer containing 6% SDS followed by boiling for 5 min. Labeled proteins were resolved by SDS-PAGE and analyzed by Coomassie blue staining and autoradiography using Phosphorimager™ plates (Molecular Dynamics, Sunnyvale, CA, USA) and a Typhoon™ imaging system (GE Healthcare).

### 2.6. Total Protein Extraction

Yeast cells were grown to mid-exponential phase, harvested by centrifugation and then stored at −80 °C before use. Cell pellets were thawed on ice and incubated in 2 vol of lysis buffer (1.85 M NaOH, 7.4% β-mercaptoethanol). To precipitate the proteins released from the lysed cells, a one-tenth volume of ice-cold 50% TCA was added. After incubation on ice for 20 min, the precipitated protein was collected by centrifugation at maximum rpm in a microfuge. After removal of the supernatant solution, the protein pellet was washed twice with chilled (−20 °C) acetone. To resuspend the precipitated protein and ensure neutralization of any residual TCA, the pellets were resuspended in 5% SDS in 0.1 M Tris base and then samples were diluted into SDS-PAGE sample buffer and analyzed by SDS-PAGE. For phosphatase treatment, TCA-precipitated proteins were solubilized in a solution constituted by mixing 42 μL of solubilization buffer (0.1 M NaCl, 0.3 M sorbitol, 25 mM MgCl_2_, 1 mM EDTA, 10 mM Tris-Cl pH 7.5,) with 18 μL of 1 M Tris base and 40 μL of 10% SDS and 2% β-mercaptoethanol. The solubilized protein was then diluted with 900 μL of 50 mM Tris-Cl (pH 8.5), to which was added 90 units of calf intestinal phosphatase (New England Biolabs, Ipswich, MA, USA), and the resulting mixture incubated for 2 h at 30 °C in the absence or presence of phosphatase inhibitor (4 mM Na_3_VO_4_). Proteins were reprecipitated again, and then resuspended and resolved by SDS-PAGE, as above.

### 2.7. Immunoblotting

Samples resolved by SDS-PAGE were transferred onto nitrocellulose membranes and blocked in Odyssey^TM^ blocking buffer or Intercept^TM^ blocking buffer + 0.2% Tween (Li-Cor Biosciences, Lincoln, NE, USA) for 1 h at room temperature, prior to immunoblotting. Membranes were then incubated with appropriate antibodies diluted in Odyssey^TM^ buffer (Li-Cor Biosciences Lincoln, NE, USA). Antibodies for detection of specific proteins used in this study were: rabbit polyclonal anti-tRFP (Evrogen AB233) (1:1000); mouse monoclonal anti-HA (6E2) (Cell Signaling Technology 2367) (1:1000); mouse monoclonal anti-FLAG (Sigma-Aldrich F1804) (1:5000); rabbit monoclonal anti-GFP (D5.1) (Cell Signaling 2956S) (1:500); rabbit polyclonal anti-Jsn1/Puf1 [14] (1:3000); and, purified rabbit polyclonal anti-Pdr5 antibodies [46] (1:50,000). Pgk1, detected with rabbit polyclonal anti-Pgk1 antibodies (1:10,000) prepared in this laboratory [47], was used as the control for equivalent loading of yeast cell extract protein. The nitrocellulose membranes were incubated with the primary antibody overnight at 4 °C. Membranes were then washed 4 times for 5 min with 1X PBS + 0.1% Tween. For the detection by secondary antibodies, either CF680 goat anti-rabbit or CF770 goat anti-mouse IgG (Biotium, Inc., Fremont, CA, USA), membranes were incubated with antibodies diluted 1:10,000 in 1X PBS + 0.1% Tween + 0.02% SDS for 30 min at room temperature. Membranes were once again washed four times for 5 min with 1X PBS + 0.1% Tween, followed by a single wash in 1X PBS. Results were visualized using an Odyssey^TM^ CLx infrared imaging system (Li-Cor Biosciences, Lincoln, NE, USA).

### 2.8. Fluorescence Microscopy

Cells were grown at 30 °C to mid-exponential phase (A_600 nm_ = 0.6), in YPD medium and washed with SCD-Trp prior to observation. Live cells were viewed under an epifluorescence microscope (Olympus, Model BH-2; Olympus America, Center Valley, PA, USA) equipped with a 100x objective, Solalight source (Lumencor, Beaverton, OR, USA), and appropriate bandpass filter (model n°49008, Chroma Technology, Rockingham, VT). Images were collected using a CMOS camera (Model Prime 95B, Photometrics, Tucson, AZ, USA).

### 2.9. RT-qPCR

Total RNA was extracted from cultures in mid-exponential phase using Lucigen MasterPure™ Yeast RNA Purification Kit, according to the manufacturer’s protocol. Where indicated, cultures were treated for 2 h with aureobasidin A (AbA) from a stock dissolved in ethanol at a final concentration of 1.8 µM or, as the control, with an equivalent volume of solvent alone, prior to harvesting. RNA was reverse transcribed using Applied Biosystems™ High-Capacity cDNA Reverse Transcription Kit, according to the manufacturer’s instructions, and qPCR was performed with Bio-Rad iTaq Universal SYBR Green Supermix on a Bio-Rad CFX96 Touch Real-Time thermal cycler. Transcript abundance was determined by normalizing the CT values of the target transcripts *(PMP3*, *PDR5, ZEO1*) to the CT values for the *PGK1* transcript in the same samples, and fold change was calculated relative to the values from Puf1^WT^ Puf2^WT^ cells. The values presented are the averages of independent biological replicates (n = 4 for Puf1^WT^ Puf2^WT^ vs. Puf1^AA^ Puf2^AA^ mutant cells; and, n = 2 for Puf1^WT^ Puf2^WT^ cells +/− AbA) in which the extracted RNA from each replicate was analyzed in triplicate. Error bars incorporate an estimate of uncertainty calculated from the standard errors of the mean of both the control and the experimental samples and, therefore, statistical significance was assessed by calculating *p*-values using two-sample *t*-tests.

### 2.10. In Vivo Dual Fluorescence Reporter Assay

The high-throughput yeast cell-based assay for genome-wide characterization of *S. cerevisiae* proteins or protein fragments that affect either mRNA stability and/or the efficiency of mRNA translation and its application have been delineated previously [44,48] and will be further described in greater detail elsewhere (Reynaud et al., manuscript in preparation). In brief, the system utilizes a dual fluorescent protein reporter arrangement, in which yeast genes or gene fragments are fused to the coding sequence for a bacteriophage stem-loop binding protein (in our case, λN). The resulting chimeric proteins are, in turn, tethered to an mRNA that expresses a yellow fluorescent protein (YFP) via multiple copies of a hairpin (in our case, *boxB*) to which the phage protein binds that are installed within the 3′-UTR of the transcript. As an internal standard, the cells also express [from the same promoter (*PGK1*) and with the same translational initiation context] a second mRNA encoding a red fluorescent protein (mCherry) with multiple copies of a different (irrelevant) hairpin (PP7) in its 3′-UTR. Cells expressing the chimeric proteins are interrogated using flow cytometry/fluorescence-activated cell sorting (FACS) to monitor the relative levels of YFP and mCherry expression in individual cells, and compared to the YFP and mCherry expression levels in otherwise identical cells expressing the phage protein fused to an irrelevant protein (Halo tag). The control phage protein fusion and the yeast protein-phage protein chimeras are tagged with the blue fluorescent protein (BFP), which allows for gating of the FACS so that only BFP-positive cells are examined.

## 3. Results

### 3.1. Puf1 and Puf2 Are Substrates of Protein Kinase Ypk1

**Figure 1:**
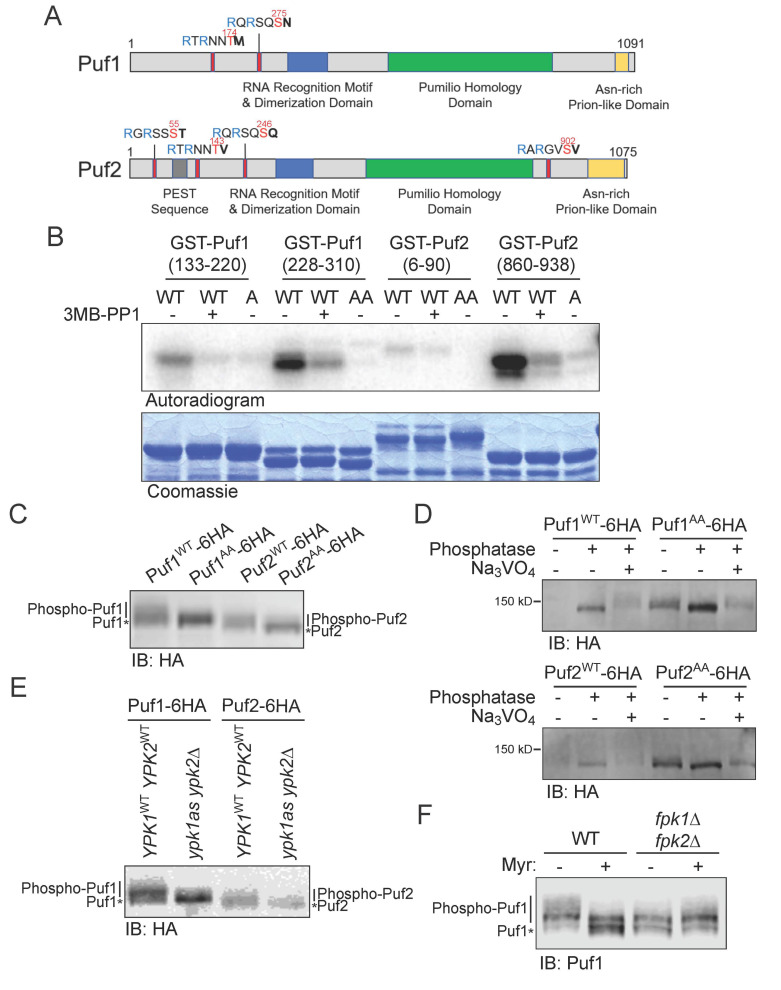
Ypk1 phosphorylates RNA-binding proteins Puf1 and Puf2. (**A**) Schematic depiction of the primary structure of *S. cerevisiae* Puf1 and Puf2. Ypk1 phosphorylation sites, *red*; RRM-like dimerization domain, *blue*; RNA-binding *Pumilio* homology domain, *green*; Asn-rich region, *yellow*; PEST-like region (rich in Pro, Glu, Ser and Thr), *gray*. (**B**) Ypk1 phosphorylates Puf1 and Puf2 in vitro at their Ypk1 sites. GST-Puf1(133–220) (pHG10), GST-Puf1(228–310) (pHG11), GST-Puf2(6–90) (pHG12), GST-Puf2(860–938) (pHG13), or the corresponding Ala substitution mutants GST-Puf1(133–220; T174A) (pHG18), GST-Puf1(228–310; S273A S275A) (pHG19), GST-Puf2(6–90; S53A S54A S55A T56A) (pHG20) and GST-Puf(860–938; S902A) (pHG21), were purified from *E. coli*, incubated with [γ-^32^P]ATP and Ypk1-as (purified from *S. cerevisiae* as described in Materials and Methods) in the absence (-) or presence (+) of 3-MB-PP1, and the resulting products resolved by SDS-PAGE and analyzed by autoradiography. Equivalent input of the substrates was confirmed by staining with Coomassie blue. (**C**) Puf1 and Puf2 are phosphorylated in vivo at their Ypk1 sites. Cells expressing Puf1^WT^-6HA (yHG15), Puf1^AA^-6HA (yHG16), Puf2^WT^-6HA (yHG17), or Puf2^AA^-6HA (yHG18) were grown to mid-exponential and total protein extracted, resolved by SDS-PAGE on 8% gels for an extended running time, and analyzed by immunoblotting with anti-HA mAb 6E2. (**D**) Ypk1 sites are major (but, not the exclusive) phosphorylation sites in Puf1 and Puf2. The same cells as in (**C**) were grown as previously. Protein extracts were treated in the absence (-) or presence (+) of calf intestinal phosphatase, in either the absence (-) or presence (+) of the phosphatase inhibitor Na_3_VO_4_, as described in Materials and Methods, resolved by SDS-PAGE as in (**C**) and analyzed by immunoblotting with anti-HA mAb 6E2. (**E**) Puf1 is phosphorylated in a Ypk1- and Ypk2-dependent manner in vivo. A wild-type strain (*YPK1^+^ YPK2^+^*) (YFR694) and its otherwise isogenic *ypk1-as ypk2*∆ derivative (YFR695) expressing Puf1^WT^-6HA, or the same strains expressing Puf2^WT^-6HA (YFR699 and YFR700, respectively) were grown to mid-exponential phase, treated for 2 h with auroeobasidin A (1.8 µM) to stimulate TORC2-Ypk1 signaling, but in the presence of 3-MB-PP1 (10 µM). Protein extracts were made, resolved, and analyzed as in (**C**). (**F**) Puf1 is also phosphorylated in a Fpk1- and Fpk2-dependent manner in vivo. A wild-type strain (BY4741) and its otherwise isogenic *fpk1*∆ *fpk2*∆ derivative (YFR205) were grown overnight in YPD, diluted into warm YPD to A_600 nm_ = 0.2, grown for 3 h, and then either treated with an equivalent volume of solvent (methanol) alone (-) or with myriocin in the same solvent (1.25 µM final concentration) (+) for 2 h. Protein extracts were made, resolved as in (**C**), and analyzed by immunoblotting with rabbit polyclonal anti-Puf1 antibodies (kind gift of Prof. Georjana Barnes, Univ. of California, Berkeley, USA).

The consensus motif for phosphorylation by Ypk1 has the sequence -R-x-R-x-x-S>T-(Hpo)-, where (Hpo) represents a preference for a hydrophobic or uncharged residue. Puf1 has two canonical Ypk1 phosphorylation sites (T174 and S275), and Puf2 has four (S55, T143, S246, and S902) (Figure 1A). Each of these sites (except T143 in Puf2) has been detected as phosphorylated in vivo in one or more published studies of the global *S. cerevisiae* phosphoproteome [49,50,51]. Although we recognized Puf1 and Puf2 as presumptive substrates of Ypk1 in our genome-wide screen [13], we did not analyze them further at the time because overexpression of Puf1 did not exhibit a genetic hallmark of established Ypk1 substrates, namely a synthetic dosage lethality (SDL) phenotype under conditions where the activity of Ypk1 is low [13]. However, in a comprehensive survey for yeast gene products that form prominent intracellular aggregates when overexpressed, both Puf1 and Puf2 were identified [52], consistent with the presence of a feature found in many prion-forming polypeptides [53], namely Asn-rich tracts at the C-terminal end of both proteins (Figure 1A; see also Figure 4A). Sequestration of overexpressed Puf1 into such aggregates, where it is inaccessible to Ypk1 and thus unable to act as a competing substrate to divert the enzyme away from its other vital targets, likely explains the lack of an SDL phenotype. Therefore, we re-initiated examination of whether Puf1 and Puf2 represent *bona fide* Ypk1 substrates.

We prepared, as GST fusions, fragments of Puf1 that cover the sequences containing the Ypk1 sites that it shares with Puf2 and fragments of Puf2 containing its two additional Ypk1 sites. These proteins were incubated with [γ-^32^P]ATP and purified Ypk1-as in the absence and presence of the inhibitor 3-MB-PP1 [13], so that the dependence of any observed phosphate incorporation could be attributed to Ypk1 itself. We found that all four proteins were phosphorylated by Ypk1 in vitro, albeit to different extents, and that, in every case, incorporation was greatly diminished in the presence of 3-MB-PP1, confirming that the phosphorylation was indeed due to Ypk1 (Figure 1B). To demonstrate that the incorporation was occurring specifically at the predicted Ypk1 sites, we conducted the same analysis using mutant versions of the GST-fusions in which the phospho-acceptor residue (Ser or Thr) was substituted with Ala (indicated with a superscript A). We have documented previously that when the Ypk1 consensus motif contains other Ser (or Thr) residues, these can be phosphorylated when the primary site is absent [20]. For that reason, in the GST-Puf1(228-310) fragment, both S273 and S275 were mutated to A; and, likewise, in the GST-Puf2(6-90) fragment, S53, S54, S55 and T56 were all mutated to A (indicated, in such cases, with an AA superscript, as a shorthand). We found that incorporation was almost completely abolished by the Ala mutations (Figure 1B), confirming that virtually all of the incorporation was occurring at the Ypk1 sites, despite the presence of numerous other Ser and Thr residues elsewhere in these Puf1 and Puf2 fragments.

To determine whether Puf1 and Puf2 are phosphoproteins in vivo, as indicated by phosphoproteome analyses [49,50,51], specifically because they are phosphorylated at their Ypk1 sites, we expressed full-length epitope-tagged derivatives of Puf1 and Puf2, and corresponding mutants (abbreviated “AA”) lacking all Ypk1 phospho-acceptor residues [Puf1(T174 S273A S275A) and Puf2(S53A S54A S55A T56A S143A S244A S246A S902A)] in otherwise wild-type (WT) yeast cells. When extracted under conditions that prevent phosphatase action (see Materials and Methods), Puf1^WT^ and Puf2^WT^ migrated as a smear of isoforms in standard SDS-PAGE, as expected for phospho-proteins, whereas the Puf1^AA^ and Puf2^AA^ mutants ran as more compact bands with a faster mobility, as expected for loss of phosphorylation (Figure 1C). Likewise, treatment of the extracted proteins in vitro with calf intestinal phosphatase caused a dramatic collapse of the Puf1^WT^ and Puf2^WT^ isoforms into a faster-mobility species, whereas this shift was less marked for the Puf1^AA^ and Puf2^AA^ mutants (Figure 1D). The mobility shift caused by phosphatase treatment of the Puf1^AA^ and Puf2^AA^ mutants, albeit modest, indicates that Puf1 and Puf2 must be phosphorylated at other positions in addition to its Ypk1 sites. Nonetheless, it is clear the Ypk1 sites are major sites of phosphorylation in Puf1 and Puf2 in vivo. Indeed, a very similar collapse of the smear of Puf1^WT^ and Puf2^WT^ isoforms was observed when the epitope-tagged proteins were expressed in cells (*ypk1-as ypk2*∆) where Ypk1 activity could be minimized by treatment with the 3-MB-PP1 inhibitor, as compared to control (*YPK1^+^ YPK2^+^*) cells that are “immune” to the effects of the inhibitor (Figure 1E), corroborating that Puf1 and Puf2 are phosphorylated in a Ypk1-dependent manner in vivo. 

We have some indication as to the identity of at least one other protein kinase that phosphorylates Puf1 and Puf2 that is also under the control of the TORC2-Ypk1 signaling axis. We have demonstrated previously that, when TORC2-Ypk1 signaling is activated by treating the cells with myriocin to cause a depletion of sphingolipids [20,54,55], a major target of activated Ypk1 is the protein kinase Fpk1 and its much less abundant paralog Fpk2/Kin82 [28] and, further, that the effect of Ypk1-mediated phosphorylation is to inhibit Fpk1 and Fpk2 [33,37]. Moreover, we have provided evidence that sphingolipids per se are required for optimal Fpk1 function [33,37]. Indeed, when endogenous Puf1 was examined in cells incubated in the absence and presence of myriocin, it was clear that in the myriocin-treated cells, in which Ypk1-mediated phosphorylation of Puf1 should be stimulated and sphingolipids depleted, some of the slowest mobility species in the smear of isoforms were eliminated and, moreover, the overall pattern of phospho-isoforms closely resembled that seen in cells lacking both Fpk1 and Fpk2 (Figure 1F). Furthermore, we note that Puf1 and Puf2 share a consensus Fpk1 phospho-acceptor site, -R-x-S>T-(Hpo)-D/E- [33,56], just upstream of their two shared Ypk1 consensus sites (and Puf1 has an additional site within its *Pumilio* homology domain) (see Figure 4A). The Fpk1 consensus site in Puf1 (S157) that it shares with Puf2 (see Figure 4A) has been detected as phosphorylated in six different global analyses of the *S. cerevisiae* phosphoproteome [49,50,51,57,58,59] Thus, our results indicate that under conditions that elevate Ypk1 activity and reduce sphingolipid content, Puf1 and Puf2 will undergo increased phosphorylation at their Ypk1 sites with a concomitant decrease in phosphorylation at their Fpk1 site(s). 

### 3.2. Phosphorylation by Ypk1 Down-Regulates the Function of Puf1 and Puf2 

**Figure 2:**
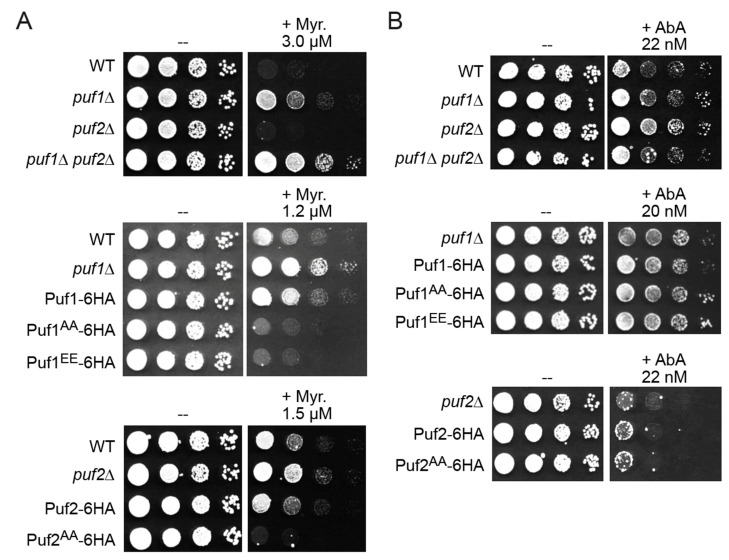
Phosphorylation down-regulates Puf1 and Puf2 function. (**A**) *Top*, overnight cultures of WT (BY4741), and derived *puf1*∆ (yHG5), *puf2*∆ (yHG4), and *puf1*∆ *puf2*∆ (yHG2) cells, were serially diluted 10-fold and plated onto YPD containing solvent alone (-) or myriocin (Myr) at the indicated concentration. Pictures were taken after two days of growth. *Middle*, overnight cultures of WT, *puf1*∆ cells (yHG5), or cells expressing Puf1-6HA (yHG15), Puf1^AA^-6HA (yHG16) or Puf1^EE^-6HA (yHG22), as indicated, were serially diluted 10-fold and plated onto YPD containing solvent alone (-) or myriocin (Myr) at the indicated concentration. Pictures were taken after two days of growth. *Bottom*, overnight cultures of WT, *puf2*∆ cells (yHG4), or cells expressing Puf2^WT^-HA (yHG17) or Puf2^AA^-HA (yHG18), as indicated, were serially diluted 10-fold and plated onto YPD containing solvent alone (-) or myriocin (Myr) at the indicated concentration. (**B**) Same as in (**A**), except the cells were exposed to solvent alone (-) or aureobasidin A (AbA) at the indicated concentration.

The steady levels of Puf1 (~2534 molecules per cell) and Puf2 (~2178 molecules per cell) are similar [28] and they bind to many of the same mRNA targets [15,17], suggesting a common function. Consistent with that conclusion, it was reported that deletion of either *PUF1* or *PUF2* increased resistance to myriocin [18]. We confirm that loss of Puf1 renders yeast cells more resistant to myriocin, compared to otherwise isogenic WT cells (Figure 2A). At the initial concentration tested (3 µM), absence of Puf2 alone did not elevate myriocin resistance; however, a *puf2*∆ mutation enhanced the myriocin resistance of *puf1*∆ cells (Figure 2A, *top panel*), indicating that Puf2 does have some shared role with Puf1.

Indeed, at lower doses (1.2–1.5 µM) of myriocin, absence of either Puf1 (Figure 2A, *middle panel*) or Puf2 (Figure 2A, *bottom panel*) permitted enhanced survival. We exploited these phenotypic differences as a means to assess how phosphorylation of Puf1 and Puf2 at their Ypk1 sites affects their function. Cells expressing Puf1^WT^-6HA from the *PUF1* promoter at its normal chromosomal locus were somewhat more resistant than otherwise isogenic WT cells expressing native Puf1, suggesting that the epitope tag is slightly deleterious to Puf1 function. Remarkably, however, the cells expressing Puf1^AA^-6HA in the same manner exhibited a degree of myriocin sensitivity even greater than that displayed by the WT cells (Figure 2A, *middle panel*), suggesting that Ypk1-mediated phosphorylation impedes Puf1 function. The same trends were observed for Puf2^WT^-6HA and Puf2^AA^-6HA strains (Figure 2A, *bottom panel*). These genetic findings strongly suggest that phosphorylation at their Ypk1 sites negatively regulates Puf1 and Puf2 function.

Killing by any antibiotic with an intracellular target requires entry of that compound into the cell. Hence, the increased resistance to myriocin of cells lacking Puf1 or Puf2 could be due to a change in the level of PM proteins whose transcripts are under Puf1 and Puf2 control, thereby affecting permeability of the PM to myriocin, or, alternatively, due to more potent upregulation of TORC2-Ypk1 signaling and an ensuing increased rate of sphingolipid biosynthesis to counteract and compensate for the inhibition of SPT by myriocin per se. To distinguish between these possibilities, we treated cells with aureobasidin A (AbA), another antifungal compound, which blocks sphingolipid biosynthesis at a later step in the pathway; AbA inhibits the enzyme (Aur1) that catalyzes formation of the first intermediate (inositol-phosphorylceramide) for synthesis of the essential end-products of the yeast pathway (complex sphingolipids) [60]. AbA is strongly growth inhibitory to yeast (at the nM level) and, like myriocin, causes potent activation of TORC2-Ypk1 signaling [39,54]. We found that *puf1*∆ and *puf2*∆ single mutants, and even a *puf1*∆ *puf2*∆ double mutant, were not reproducibly more resistant to AbA (Figure 2B, *top panel*), and whether or not Puf1 (Figure 2B, *middle panel*) or Puf2 (Figure 2B, *bottom panel*) was susceptible to Ypk1-mediated phosphorylation had little or no effect. These results suggest that absence of Puf1 and Puf2 (or both) confers myriocin resistance by specific effects on permeability of the cell to this particular drug rather than via effects on the strength of TORC2-Ypk1 signaling per se.

A Puf1^EE^-6HA mutant [Puf1(T174E S273E S275E)-6HA] displayed the same phenotype as the Puf1^AA^-6HA mutant (Figure 2A, *middle panel,* and Figure 2B, *middle panel*), indicating that, in the case of this particular protein and these particular sites, Ser/Thr-to-Glu mutations are not an adequate mimic for authentic phosphate groups, as has been observed for other proteins whose function is controlled by phosphorylation both by us [32] and others [61]. 

### 3.3. Ypk1 Phosphorylation Promotes Protein Production from Puf1- and Puf2-Bound mRNAs

**Figure 3:**
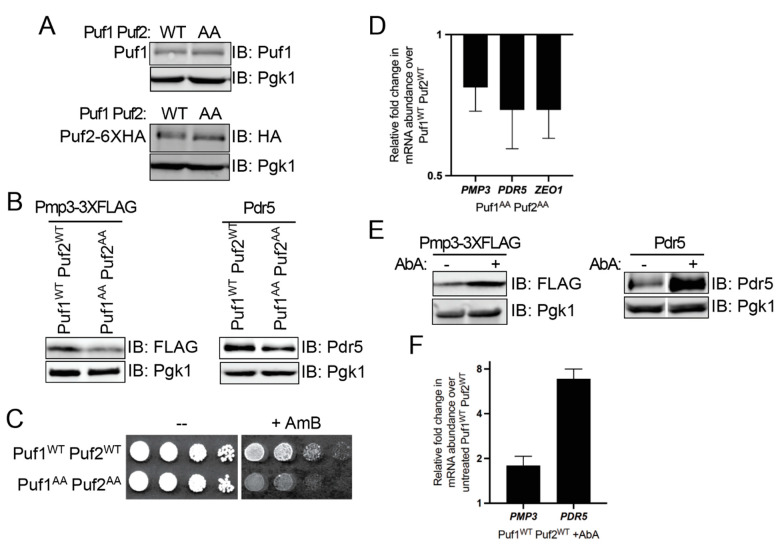
Phosphorylation of Puf1 and Puf2 increases the level of the protein products of their target mRNAs by impeding their ability to promote degradation of the same transcripts. (**A**) Cells expressing Puf1^WT^ Puf2^WT^-6HA (YFR739-A) or Puf1^AA^ Puf2^AA^-6HA (YFR738) were grown to mid-exponential phase, total protein extracted, resolved by standard SDS-PAGE on 10% gels and analyzed by immunoblotting. (**B**) As in (**A**), except that strains Puf1^WT^ Puf2^WT^-6HA Pmp3-3xFLAG (ySP18) and Puf1^AA^ Puf2^AA^-6HA Pmp3-3xFLAG (ySP19) were used for measuring the protein levels of Pmp3-3xFLAG and Puf1^WT^ Puf2^WT^-6HA (YFR739-A) and Puf1^AA^ Puf2^AA^-6HA (YFR738) were used for measuring the protein levels of Pdr5. (**C**) Overnight cultures of the same cells as in (**A**) were serially diluted 10-fold and plated onto YPD containing solvent (DMSO) alone (-) or an equivalent volume of the same solvent containing AmB (0.6 µM final concentration). (**D**) RT-qPCR analysis (n = 4) of *PMP3*, *PDR5,* and *ZEO1* transcript abundance in Puf1^AA^ Puf2^AA^-6HA (YFR738) cells compared to the abundance in Puf1^WT^ Puf2^WT^-6HA (YFR739-A) controls (set as [1]). All mRNA expression levels were normalized to the amount of the transcript for the glycolytic enzyme *PGK1* in the same RNA sample. Whisker bars indicate standard error estimated as described in Materials and Methods. All of the changes were statistically significant; *p*-values were: *PMP3, p*=0.028; *PDR5, p* = 0.048; and, *ZEO1*, *p* = 0.034. (**E**) Puf1^WT^ Puf2^WT^-6HA Pmp3-3xFLAG (ySP18) and Puf1^WT^ Puf2^WT^-6HA (YFR739-A) cells were grown to mid-exponential phase in YPD, diluted to A_600 nm_ = 0.2 in warm YPD, grown for 3 h and then treated with vehicle (EtOH) alone (-) or AbA (1.8 µM final concentration) for 2 h. Protein extracts were made, resolved by SDS-PAGE and analyzed by immunoblotting as in (**B**). (**F**) RT-qPCR analysis (n = 2), as in (**D**), of *PMP3* and *PDR5* transcript abundance in Puf1^WT^ Puf2^WT^-6HA (YFR739-A) cells treated with Aureobasidin A (AbA) compared to the abundance in the untreated control (set as [1]). The changes were statistically significant; *p*-values were: *PMP3*, *p* = 0.028; and, *PDR5, p* = 0.007.

To further explore at the biochemical level the above genetic findings suggesting that phosphorylation at their Ypk1 sites negatively regulates Puf1 and Puf2 function, we examined the steady-state level of protein products of transcripts characterized as direct targets of Puf1 and Puf2. Toward this end, we constructed a pair of strains that express from their native promoters at their normal chromosomal loci either Puf1^WT^ and Puf2^WT^-6HA or Puf1^AA^ and Puf2^AA^-6HA. Puf1 mRNA itself is reportedly a target for Puf1 and Puf2 [15,17]. Hence, to construct these strains and eliminate any possible auto-regulation, we inserted a *LEU2* marker just after the stop codon of the *PUF1* and *PUF2* ORFs to sever the native 3′-UTRs from the resulting *PUF1* and *PUF2* coding sequences, and confirmed by immunoblotting that both the WT and AA versions of both proteins were expressed at an equivalent level in the two strains (Figure 3A, *top and bottom panels*). 

We used this pair of strains to look, first, at Pmp3, a highly conserved, very small protein (55 residues) with two, strongly predicted, hydrophobic transmembrane segments [62] because multiple groups had corroborated the initial report [15] that *PMP3* mRNA was bound by Puf1 and Puf2 [17,63,64]. To monitor Pmp3, a FLAG tag was appended in-frame to its coding sequence without altering or removing its native 3′-UTR using Cas9-mediated gene engineering [65,66]. We found that, compared to the amount of the glycolytic enzyme Pgk1 as a control for equivalent loading, there was a readily detectable decrease in the level of Pmp3 in the cells expressing Puf1 and Puf2 lacking their Ypk1 sites compared to its level in WT cells (Figure 3B, *left panel*). We corroborated this conclusion using a genetic approach. It has been shown that cells lacking Pmp3 are hypersensitive to the growth inhibitory action of the ergosterol-binding polyene antibiotic amphotericin B (AmB) [67,68]. We reasoned that a reduction in Pmp3 should likewise cause an increase in sensitivity to AmB. Indeed, as expected and consistent with a lower level of Pmp3, the cells expressing Puf1 and Puf2 lacking their Ypk1 sites were more sensitive to AmB than otherwise isogenic cells expressing WT Puf1 and Puf2 (Figure 3C).

We next examined Pdr5, another integral PM protein whose transcript is a Puf1 and Puf2 target [15,17]. Pdr5 is a large (1511 residues) multi-drug transporter in the ABC-family with 12 transmembrane segments [69]. To monitor Pdr5, we utilized a rabbit polyclonal antibody against native Pdr5, generously provided by Prof. Karl Kuchler (Medical University of Vienna, Austria). As with Pmp3, we found that, compared to the amount of the glycolytic enzyme Pgk1 as a control for equivalent loading, there was a modest decrease in the level of Pdr5 in the cells expressing Puf1 and Puf2 lacking their Ypk1 sites compared to its level in WT cells (Figure 3B, *right panel*).

These results indicate that, when Puf1 and Puf2 cannot be phosphorylated by Ypk1, production of the protein products of the transcripts to which they bind is reduced. This post-transcriptional repression could be exerted by impeding the efficiency of translation of each existing mRNA to which Puf1 and/or Puf2 is bound with no change in its level, or could be due to a reduction in the level of the cognate mRNA because binding of Puf1 and/or Puf2 is destabilizing. Indeed, there is evidence that association of Puf family proteins, including yeast Puf1 [63,70] and Puf3 [71,72], enhances the rate of decay of the transcripts to which they bind.

To distinguish between these possibilities, we extracted RNA from the same strains and carried out RT-qPCR analysis of the *PMP3* and *PDR5* transcripts. We found that, indeed, relative to the cells expressing WT Puf1 and Puf2, the cells expressing Puf1 and Puf2 lacking their Ypk1 sites reproducibly contained a lower level of both transcripts (Figure 3D). To further solidify that conclusion, we also examined by RT-qPCR the level of the *ZEO1* transcript because it is another well-validated target of Puf1 and Puf2 [70]. Zeo1 is a peripheral PM protein that is tightly associated with Mid2, a heavily O-glycosylated integral PM protein with a single transmembrane segment [73]. As for the *PMP3* and *PDR5* transcripts, we found that, relative to the WT (control) cells, the level of the *ZEO1* mRNA was reproducibly lower in the cells expressing Puf1 and Puf2 lacking their Ypk1 sites (Figure 3D). 

These findings suggest that, in the absence of Ypk1 phosphorylation, Puf1 and Puf2 are more efficient in promoting turnover of the transcripts to which they bind. That model predicts, conversely, that elevating Ypk1-mediated phosphorylation of Puf1 and Puf2 should impede Puf1- and Puf2-promoted transcript decay and raise the steady-state level of the cognate products of those mRNAs. Indeed, when we treated WT cells with AbA to stimulate TORC2-Ypk1 phosphorylation, the steady levels of both the Pmp3 and Pdr5 proteins rose markedly (Figure 3E), concomitant with an increase in the level of both of the cognate transcripts (Figure 3F), when compared to control cells not treated with AbA. Thus, Ypk1 phosphorylation relieves Puf1- and Puf2-mediated translational repression mainly by counteracting their negative effect on transcript stability.

### 3.4. Additional Mechanistic Insight from Application of a Heterologous Protein-RNA Tethering and Fluorescent Protein Reporter Assay

In prior work, a genome-wide, single-cell, flow cytometry (FACS)-based screen for proteins and protein fragments able to exert post-transcriptional repression in yeast was devised and implemented [44,48]. As described in greater detail in Materials and Methods, in this approach, the mRNA encoding a fluorescent reporter protein (YFP) contains multiple hairpins inserted in its 3′-UTR. The proteins and protein fragments to be tested are fused to a bacteriophage stem-loop-binding protein and thus tethered to the transcript by binding of the fusion protein to the hairpins. In this assay format, the phage protein fusions also are marked with BFP, which allows for gating of the FACS so that only BFP-positive cells are examined. In the same cells, a second mRNA encoding a red fluorescent protein (here abbreviated RFP) is expressed from the same promoter and with the same translational start context, but with irrelevant hairpins in its 3′-UTR, and serves as an internal standard (control). A culture is interrogated using FACS to monitor the relative levels of YFP and RFP expression in individual BFP-positive cells.

**Figure 4:**
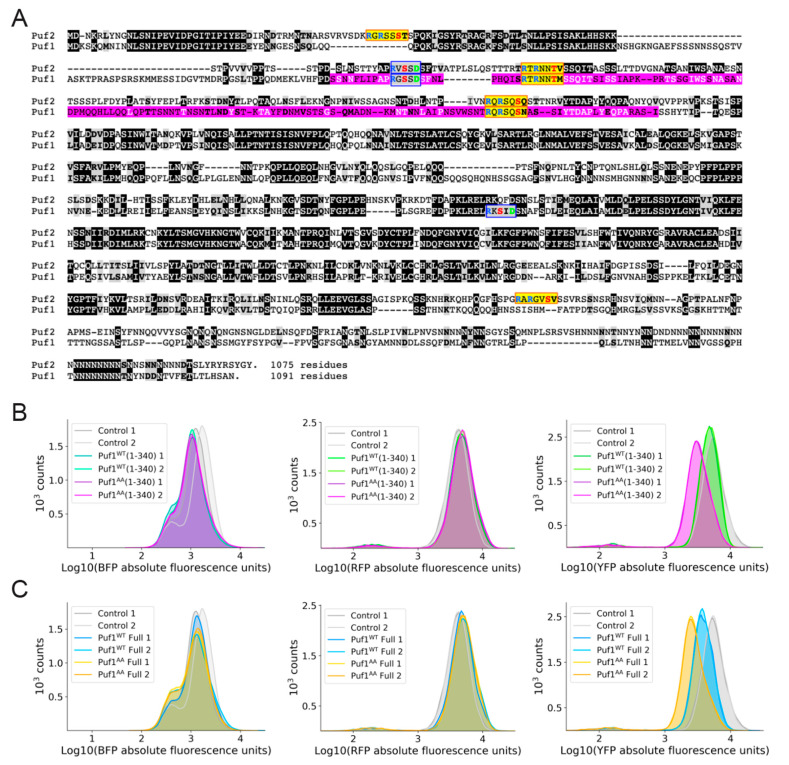
Absence of Ypk1-mediated phosphorylation enhances transcriptional repression by Puf1. (**A**) Sequence alignment of Puf1 (*bottom*) and Puf2 (*top*). Identities, *white letter on a black box*; standard conservative substitutions, *bold letter in a gray box*; consensus Ypk1 phosphorylation sites, *yellow boxes*; consensus Fpk1 phosphorylation sites, *blue boxes*; Puf1 fragment (residues 143-295) isolated in initial screen [45], *pink shading*. (**B**) FACS analysis of the level of expression of BFP (*left panel*), RFP (*middle panel*), and YFP (*right panel*) for control cultures expressing a Halo tag-λN-BFP fusion (*grey peaks*; n = 2), cultures expressing the Puf1^WT^(1-340)-λN-BFP fusion (*green peaks*; n = 2), and cultures expressing the Puf1^AA^(1-340-λN-BFP fusion (*violet peaks*; n = 2). (**C**) FACS analysis of the level of expression of BFP (*left panel*), RFP (*middle panel*), and YFP (*right panel*) for control cultures expressing a Halo tag-λN-BFP fusion (*grey peaks*; n = 2), cultures expressing the Puf1^WT^-λN-BFP fusion (*blue peaks*; n = 2), and cultures expressing the Puf1^AA^-λN-BFP fusion (*orange peaks*; n = 2).

Using the method just described, a fragment of Puf1 (residues 143-295) (Figure 4A) was recovered [44]. To corroborate the effect of the Puf1 fragment initially isolated, a somewhat larger N-terminal fragment of Puf1, residues 1-340 was fused to the phage protein, without and with its Ypk1 sites mutated to Ala, and compared to control cells in which an irrelevant protein (Halo tag) is fused to the phage protein. Equivalent number of BFP-positive cells were present in all three cultures (Figure 4B, *left panel*) and equivalent amounts of RFP were produced in the BFP-positive cells from all three cultures (Figure 4B, *middle panel*), but, compared to the control cells, cells expressing the Puf1^WT^(1-340)-phage protein fusion produced slightly less YFP, whereas the decrease in YFP was more pronounced in the cells expressing the Puf1^AA^(1-340)-phage protein fusion (Figure 4B, *right panel*). We next tested fusions of full-length Puf1, without and with its Ypk1 sites mutated to Ala. Once again, the same trends were observed, but were even more obvious. Equivalent number of BFP-positive cells were present in all three cultures (Figure 4C, *left panel*) and equivalent amounts of RFP were produced in the BFP-positive cells from all three cultures (Figure 4C, *middle panel*), but, compared to the control cells, cells expressing the Puf1^WT^-phage protein fusion clearly produced less YFP, and the decrease in YFP was even more pronounced in the cells expressing the Puf1^AA^-phage protein fusion (Figure 4C, *right panel*). Presumably, as we observed for the *PMP3*, *PDR5* and *ZEO1* transcripts, the lower level of YFP produced in the Puf1^AA^ cells is due to a lower level of the corresponding transcript because, without Ypk1 phosphorylation, Puf1 is better able to promote its decay, although we did not extract RNA from these cultures and test that supposition directly.

Thus, as with the endogenous Pmp3 and Pdr5 proteins, YFP (the product of a transcript to which Puf1 was artificially tethered) was expressed at a distinctly lowered level when Puf1 could not be phosphorylated by Ypk1. In this tethering assay system, the Puf1 protein or its fragments are tightly bound via the stem-loop binding protein-RNA interaction at multiple hairpins, making it unlikely that, in this context, Puf1 is able to dissociate fully from the transcript, even when phosphorylated (and we know that Puf1^WT^ is phosphorylated at its Ypk1 sites). Because cells expressing Puf1^WT^ produced more YFP than cells expressing Puf1^AA^ in this assay context, we suggest that Ypk1 phosphorylation need not obligatorily cause dissociation of Puf1 to reduce its ability to promote decay of the mRNA to which it is bound. More likely, Puf1 recruits other factors that execute the steps needed for mRNA turnover and Ypk1-mediated phosphorylation may interfere with the ability of Puf1 to recruit those factors.

### 3.5. Differential Subcellular Localization of Puf1 and Puf2 

**Figure 5:**
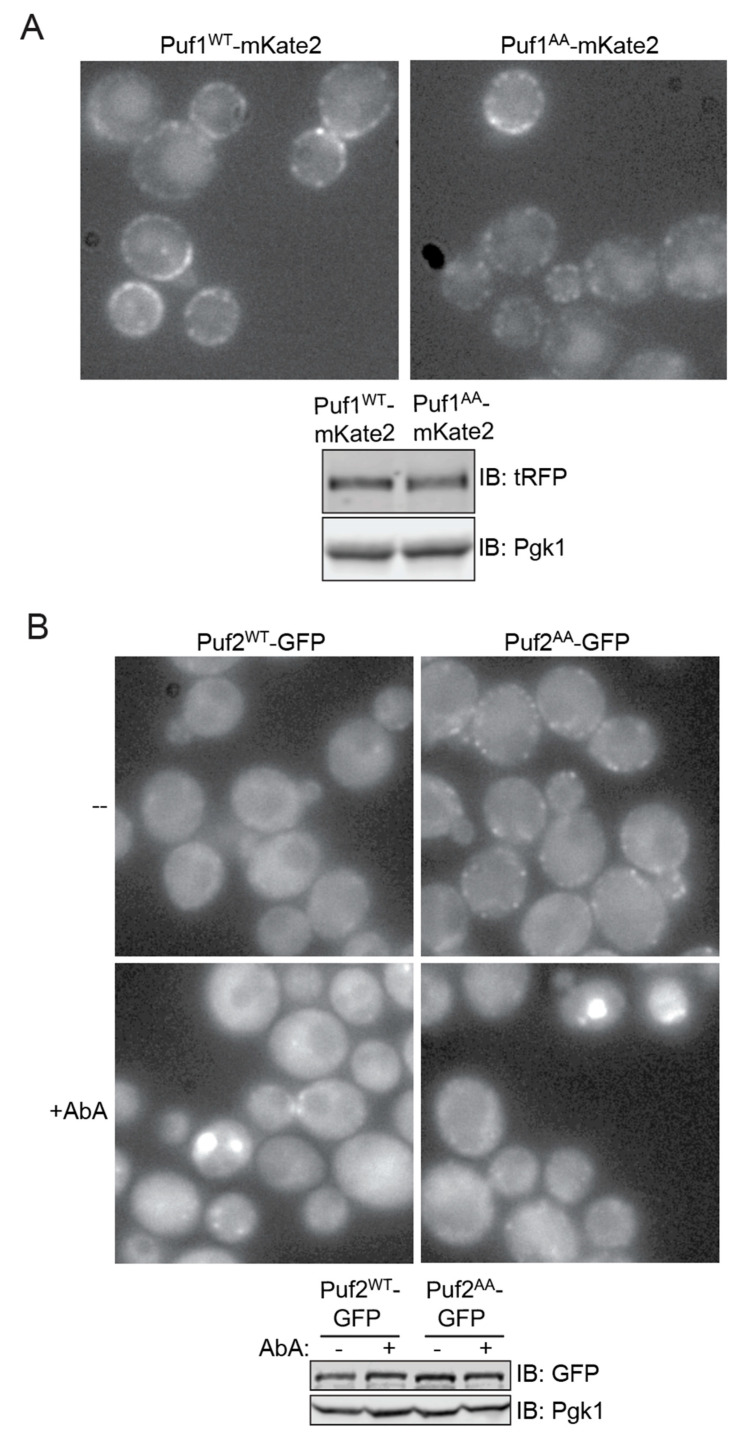
Differential localization of Puf1 and Puf2 (**A**) Strains expressing Puf1^WT^-mKate2 (yHG8) and Puf1^AA^-mKate2 (ySP1) were grown to mid-exponential phase in YPD, washed once in SCD-Trp, and viewed under an epifluorescence microscope (*upper panels*). Extracts of the same cells were prepared and samples resolved by SDS-PAGE and analyzed by immunoblotting (*lower panel*). (**B**) Strains expressing Puf2^WT^-6HA-eGFP (ySP8) and Puf2^AA^-6HA-eGFP (ySP9) were grown to mid-exponential phase in YPD, diluted to A_600 nm_ = 0.2 in warm YPD, grown for 3 h, treated with vehicle (EtOH) or AbA (1.8 µM) for 2 h, as indicated, washed once in SCD-Trp and then examined by fluorescence microscopy (*upper panels*). Extracts of the same cells were prepared and samples resolved by SDS-PAGE and analyzed by immunoblotting (*lower panel*). Haploid strains of *S. cerevisiae*, as shown here, have a mother cell diameter of ~4 µm.

To further explore how Ypk1-mediated phosphorylation affects Puf1 and Puf2, we tagged both WT and AA derivatives of each protein with a fluorescent marker protein and expressed them from their native promoters at their endogenous chromosomal loci to examine their level and distribution in live cells. In agreement with previous work using fixed cells and indirect immunofluorescence with anti-Puf1 antibodies [14], we found that Puf1^WT^-mKate2 localizes to prominent punctate bodies closely apposed to the PM (Figure 5A, *upper panel, left side*). The same has been observed for Puf1-GFP [16], providing reassurance that presence of a fluorescent marker protein does not perturb proper localization. We also found that Puf1^AA^-mKate2 displayed an indistinguishable pattern (Figure 5A, *upper panel, right side*). Moreover, Puf1^WT^-mKate2 and Puf1^AA^-mKate2 were expressed at an equivalent level (Figure 5A, *lower panel*). Thus, the absence or presence of Ypk1 phosphorylation does not influence the stability or localization of Puf1. 

In marked contrast to Puf1^WT^-mKate2, we found that Puf2^WT^-GFP was largely confined to the cytoplasm (Figure 5B, *upper left panel*), as has been reported before [16]. Strikingly, however, we found that Puf2^AA^-GFP localized to peripheral puncta closely associated with the PM (Figure 5B, *upper right panel*), resembling those observed for Puf1-mKate2. As judged by immunoblotting, the steady-state level of Puf2^AA^-GFP was similar to that for Puf2^WT^-GFP (Figure 5B, *lower panel*). Thus, Ypk1 phosphorylation appears to cause dramatic dispersal of Puf2 from cortical sites without markedly affecting its overall level.

To acutely elevate TORC2-Ypk1 signaling, we treated cells expressing Puf2^WT^-GFP and those expressing Puf2^AA^-GFP with AbA. Under this condition, in both cultures, the bulk of both Puf2^WT^-GFP and Puf2^AA^-GFP accumulated in both small and large intracellular bodies (Figure 5B, *lower panel, left and right*) that resemble P-bodies, stress granules, and other classes of protein-RNA condensates formed by liquid-like phase separation that have been observed in yeast [74,75]. Because this behavior was observed for both Puf2^WT^-GFP and Puf2^AA^-GFP, this shift cannot be attributed to direct phosphorylation of Puf2 by Ypk1 per se. However, this behavior could reflect loss of Fpk1-mediated phosphorylation in both Puf2^WT^-GFP and Puf2^AA^-GFP, given that stimulation of TORC2-Ypk1 signaling inhibits Fpk1 function.

In any event, these structures do not appear to be canonical P-bodies because when we co-expressed an mCherry-tagged derivative of a known P-body component Dcp2 [76] and treated them with AbA, the red and green fluorescent bodies observed were not congruent (data not shown). It will be interesting to test whether the Puf2-containing agglomerations are a unique structure or correspond to one of the other types of cytoplasmic protein-RNA bodies that have been described in yeast and other eukaryotic cells to date [77,78].

## 4. Discussion

As we have documented here, Puf1 and Puf2 are authentic and physiologically relevant cellular targets of the protein kinase Ypk1. We have shown further here that the effect of Ypk1-mediated phosphorylation is to negatively regulate Puf1 and Puf2 and to enhance production of the protein products encoded by the transcripts to which they bind. Finally, we have demonstrated that Ypk1 seems to achieve this up-regulation by interfering with the ability of Puf1 and Puf2 to promote turnover of the mRNAs to which they bind, consistent with other evidence that association of Puf1 and Puf2 with their client transcripts enhances their rate of decay [63,70].

Among the yeast Puf family members, Puf1 and Puf2 have certain unique features. First, as determined for the *S. pombe* Puf1 and Puf2 orthologs from crystal structures [79] and from studies of the RNA binding specificity of *S. cerevisiae* Puf1 and Puf2 [17,64,80], their *Pumilio* homology domain binds a UAAU motif, whereas that in other Puf family members typically recognizes a UGUA motif. Second, their upstream RNA recognition motif (RRM)-like fold, despite that descriptor, does not bind RNA, but rather serves as a dimerization domain. Third, the preferred binding site for Puf1 in its target mRNAs is actually a tandem UAAU-x_3-4_-UAAU motif because the RRM-mediated dimerization of Puf1 allows the *Pumilio* homology domain in each subunit to bind to one of the two UAAU sites, thus greatly favoring binding to such dual UAAU motifs over a single UAAU. It is noteworthy, in this regard, that the two Ypk1 sites (and the one Ypk1-regulated Fpk1 site) conserved in both Puf1 and Puf2 all lie just upstream of their RRM. Hence, it is possible that one way that changes in Ypk1-mediated phosphorylation at these sites negatively regulate Puf1 and Puf2 is by weakening dimer formation, thereby reducing binding affinity for UAAU-x_3-4_-UAAU-containing transcripts. However, using the protein tethering and dual fluorescent reporter assay, we found that N-terminal fragments of Puf1 that contain the Ypk1 (and Fpk1) sites that it shares with Puf2, but do not contain its RRM (residues 340-441), were sufficient to exert down-regulation of protein expression and were more potent in doing so when their Ypk1 phosphorylation sites were absent, suggesting that interference with dimerization is not (or not the only) mechanism by which phosphorylation negatively regulates Puf1 function.

For these reasons, we favor a model in which Ypk1-mediated phosphorylation interferes with the ability of Puf1 and Puf2 to associate with factors responsible for executing reactions necessary to initiate mRNA decay, consistent with substantial evidence that other Puf family members in yeast and other organisms recruit the CCR4–POP2–NOT deadenylase complex for poly(A) tail removal (reviewed in [81]). Indeed, regulated degradation of mRNAs often acts in concert with regulated transcription to dictate mRNA abundance and, thereby, to adjust the rate of production of the cognate gene products at a level optimal to meet the prevailing conditions [82,83]. In fact, the intertwined effects of transcriptional and translational control may explain why, in our hands, the TORC2-Ypk1 stimulated upregulation of one Puf1 and Puf2 target (Pdr5) was so much more dramatic than for another Puf1 and Puf2 target (Pmp3). In any event, at least in yeast, Puf1 and Puf2 are prominent examples of RNA-binding proteins that coordinate an extensive post-transcriptional gene expression program; they associate with and, as we have shown here, modulate the level of transcripts that are members of a large group of mRNAs that encode manifold proteins located within or associated with the yeast PM [15,17,70].

Pmp3, one of the Puf1 and Puf2 targets we analyzed, is a small highly hydrophobic protein that shows remarkable cross-kingdom conservation, from bacteria to plants [62,84,85]. *S. cerevisiae* cells lacking Pmp3 are hypersensitive to the growth inhibitory action of AmB [67,68]. As we showed here, if Puf1 and Puf2 cannot be phosphorylated by Ypk1, a condition we demonstrated lowers the amount of Pmp3 and the *PMP3* transcript, it increases the sensitivity of cells to AmB, as expected. Another means to prevent efficient Ypk1-mediated Puf1 and Puf2 phosphorylation and, thus, would be expected to lower Pmp3, is to eliminate Ypk1 itself. Indeed, such a reduction in Pmp3 may be a contributing factor to our previous observation that *ypk1*∆ mutants are significantly more sensitive to AmB than otherwise isogenic WT cells [34]. Likewise, we have found (data not shown) that cells expressing an allele of Fpk1, Fpk1^11A^, that is immune to phosphorylation by another protein kinase (Gin4) that, like Ypk1, negatively regulates Fpk1 function [86], are also distinctly more sensitive to AmB than otherwise isogenic WT cells, suggesting that Pmp3 level may be lower in cells with elevated Fpk1 activity. Thus, phosphorylation of Puf1 and Puf2 at their conserved N-terminal consensus Fpk1 site may *enhance* Puf1- and Puf2-mediated translational repression. Given this suggestion that the effects of Ypk1 and Fpk1 phosphorylation have opposing effects on the translation-repressing functions of Puf1 and Puf2, it makes physiological sense that stimulation of TORC2-Ypk1 signaling would promote Ypk1-mediated phosphorylation of Puf1 and Puf2 and, simultaneously (through Ypk1-imposed inhibition of Fpk1) reduce Fpk1-mediated phosphorylation of Puf1 and Puf2. In this way, activation of TORC2-Ypk1 signaling would more efficiently upregulate production of the protein products of the transcripts under Puf1 and Puf2 control. At least for Puf2, the lack of Ypk1 phosphorylation has a dramatic effect on its subcellular localization, whereas the same was not observed for Puf1. In subsequent studies, it will be interesting to explore the molecular basis for why Puf1 and Puf2 differ in this respect. A related question is the Ypk1 site-independent mechanism by which depletion of complex sphingolipids in AbA-treated cells elicits the formation of the intracellular Puf2 aggregates we observed. This behavior is reminiscent of the finding that, when hypophosphorylated, yeast Puf3 becomes trapped in intracellular RNA-containing foci under conditions of glucose starvation [87].

Because Puf1 and Puf2 control such an extended network of PM components, it might be anticipated that alterations in the functions of these proteins would impact PM structure and function in unanticipated ways. For example, we showed here, as have others [18], that cells lacking Puf1 and Puf2 are more resistant to the growth-inhibitory action of Myr than WT cells, likely due to less efficient entry of Myr into the mutant cells. Similarly, it has been reported that cells lacking Puf2 exhibit increased resistance to cycloheximide and paromomycin [88], again presumably due to changes in PM composition that affect the ability of these compounds to enter cells. Yet, for other molecules, such as AbA, we observed no significant difference in the sensitivity to this antibiotic between WT cells and *puf1*∆, *puf2*∆, and even *puf1*∆ *puf2*∆ mutants.

## 5. Conclusions

The most important conclusion of the work presented here is that phosphorylation of Puf1 and Puf2 by the protein kinase Ypk1 is yet another mechanism by which the TORC2-Ypk1 signaling axis controls the composition and function of the PM. In this sense, the regulatory circuitry we have described here joins a growing list of examples which illustrate that phosphorylation is a widely used means for regulating the function of Puf family members [87,89,90] and other classes of mRNA-binding proteins [91,92,93].

## Figures and Tables

**Table 1:** *S. cerevisiae* strains used in this study.

Scheme	Genotype	Source or Reference
BY4741	*MATahis3*∆*1 leu2*∆*0 ura3*∆*0 met15*∆*0*	Research Genetics, Inc.
BY4742	*MATα his3*∆*1 leu2*∆*0 ura3*∆*0 lys2*∆*0*	Research Genetics, Inc.
yAM135-A	BY4741 Ypk1(L424A)::*ura3*^-^ *ypk2*∆::KanMX4	[13]
yHG2	BY4742 *puf1*∆::KanMX *puf2*∆::KanMX	Research Genetics, Inc.
yHG4	BY4742 *puf2*∆::KanMX	Research Genetics, Inc.
yHG5	BY4742 *puf1*∆::KanMX	This study
yHG8	BY4742 Puf1-mKate2::*SpHIS5*	This study
ySP1	BY4742 Puf1(T174A S275A)-mKate2::*SpHIS5*	This study
ySP8	BY4742 Puf2-6HA-eGFP::*SpHIS5-LEU2*	This study
ySP9	BY4742 Puf2(S53A S54A S55A T56A T143A S246A S902A)-6HA-eGFP::*SpHIS5-LEU2*	This study
yHG15	BY4742 Puf1-6HA*::LEU2*	This study
yHG16	BY4742 Puf1(T174A S273A S275A)-6HA::*LEU2*	This study
yHG22	BY4742 Puf1(T174E S273E S275E)-6HA::*LEU2*	This study
yHG17	BY4742 Puf2-6HA::*LEU2*	This study
yHG18	BY4742 Puf2(S53A S54A S55A T56A T143A S246A S902A)-6HA::*LEU2*	This study
YFR694	BY4741 Puf1-6HA::*LEU2 lys2*∆*0 MET15*	This study
YFR695	BY4741 Puf1-6HA::*LEU2* Ypk1(L424A)::*URA3 ypk2*∆::KanMX4	This study
YFR699	BY4741 Puf2-6HA::*LEU2*	This study
YFR700	BY4741 Puf2-6HA::*LEU2* Ypk1(L424A)::*URA3 ypk2*∆::KanMX4	This study
ySP18	BY4742 Puf1::*LEU2* Puf2-6HA::*LEU2* Pmp3-3XFLAG *met15*∆*0*	This study
ySP19	BY4742 Puf1(T174A S273A S275A)::*LEU2* Puf2(S53A S54A S55A T56A T143A S246A S902A)-6HA::*LEU2* Pmp3-3XFLAG	This study
YFR205	BY4741 *fpk1*∆::KanMX *fpk2*∆::KanMX *lys2*∆*0*	[33]
YFR739-A	BY4742 Puf1::*LEU2* Puf2-6HA::*LEU2 met15*∆*0*	This study
YFR738	BY4742 Puf1(T174A S273A S275A)::*LEU2* Puf2(S53A S54A S55A T56A T143A S246A S902A)-6HA::*LEU2*	This study

**Table 2:** Plasmids used in this study.

Plasmid	Description	Source/Reference
pAX50	pBG1805 *GAL1_prom_*-Ypk1(L424A), *URA3*	[13]
pGEX4T-1	GST tag, bacterial expression vector, Amp^R^	GE Healthcare, Inc.
pHG10	pGEX4T-1 Puf1(133-220)	This study
pHG11	pGEX4T-1 Puf1(228-310)	This study
pHG12	pGEX4T-1 Puf2(6-90)	This study
pHG13	pGEX4T-1 Puf2(860-938)	This study
pHG18	pGEX4T-1 Puf1(133-220) (T174A)	This study
pHG19	pGEX4T-1 Puf1(228-310) (S273A S275A)	This study
pHG20	pGEX4T-1 Puf2(6-90) (S53A S54A S55A T56A)	This study
pHG21	pGEX4T-1 Puf2(860-938) (S902A)	This study
pKS137	*CEN*, *PGK1_prom_*-λN-1XFLAG-BFP::SpHIS5	[44]
pHG32	pKS137-Puf1-λN-1XFLAG-BFP	This study
pHG33	pKS137-Puf1(T174A S275A)-λN-1XFLAG-BFP	This study
pHG35	pKS137-Puf1(1-340)-λN-1XFLAG-BFP	This study
pHG36	pKS137-Puf1(1-340) T174A S275A-λN-1XFLAG-BFP	This study
pNTI473	pPGK1::mCherry::(PP7)_3_	[44]
pGF-V789	pRS316-*GAL_prom_*-Cas9	[45]
pSP9	pRS423-sgRNA*^PMP3^*	This study

## Data Availability

Not applicable.
